# Early-onset of type 2 diabetes mellitus is a risk factor for diabetic nephropathy progression: a biopsy-based study

**DOI:** 10.18632/aging.202624

**Published:** 2021-03-03

**Authors:** Yucheng Wu, Yiting Wang, Junlin Zhang, Rui Zhang, Lijun Zhao, Honghong Ren, Yutong Zou, Tingli Wang, Jiali Wang, Yuancheng Zhao, Chunmei Qin, Huan Xu, Lin Li, Zhonglin Chai, Mark E. Cooper, Nanwei Tong, Fang Liu

**Affiliations:** 1 Division of Nephrology, West China Hospital of Sichuan University, Chengdu, Sichuan, China; 2 Laboratory of Diabetic Kidney Disease, Centre of Diabetes and Metabolism Research, West China Hospital of Sichuan University, Chengdu, Sichuan, China; 3 Division of Pathology, West China Hospital of Sichuan University, Chengdu, Sichuan, China; 4 Department of Diabetes, Central Clinical School, Monash University, Melbourne, Australia; 5 Division of Endocrinology, West China Hospital of Sichuan University, Chengdu, Sichuan, China

**Keywords:** diabetic nephropathy, early-onset, T2DM

## Abstract

Several studies show that patients with early-onset diabetes have higher risk of diabetic complications than those diagnosed in middle age. However, whether early-onset of type 2 diabetes mellitus (T2DM) is a risk factor for diabetic nephropathy (DN) progression remains unclear, especially a lack of data in biopsy-confirmed cohort. In This study, we enrolled 257 patients with T2DM and biopsy-confirmed DN to investigate the role of early-onset T2DM in DN progression. Participants were divided into two groups according to the age of T2DM diagnosis: early-onset group (less than 40 years) and later-onset group (40 years or older). We found that patients with early-onset T2DM had higher glomerular grades and arteriolar hyalinosis scores than those in later-onset group. After adjusted for confounding factors, early-onset of T2DM remained an independent predictor of end-stage renal disease (ESRD) for patients with DN. In conclusion, although with the comparable renal function and proteinuria, patients with early-onset T2DM and DN had worse renal pathological changes than those with later-onset. Early-onset of T2DM might be an important predictor of ESRD for patients with DN, which called more attention to early supervision and prevention for patients with early-onset T2DM and DN.

## INTRODUCTION

During the past decades, the prevalence of diabetes mellitus (DM) has been increasing around the world, more than 90% of which were type 2 diabetes mellitus(T2DM) [1]. As one of the major microvascular complication, diabetic nephropathy (DN) is the leading cause of end stage renal disease (ESRD) in developed countries as well as in some developing countries, which causes huge healthy and economic burden worldwide [2, 3]. Several factors have been clarified to be related to DN progression, such as hyperglycemia, hypertension, dyslipidemia as well as the DN duration [4]. The impact of onset age of T2DM on DN remains to be investigated.

Although much attention has been paid on older patients with T2DM, young people suffered from T2DM was not well recognized. The age of patients with T2DM is falling and the incidence of early-onset T2DM has been rising rapidly in recent years [5]. For these patients, early-onset of T2DM meant longer exposure of metabolic disorders and higher risk for chronic diabetic complications [6]. Some previous studies showed that compared with those diagnosed in older people, patients with early-onset T2DM might have higher risk of albuminuria progression and ESRD [7, 8]. However, DN is a complex disease with high degree of heterogeneity and various clinic manifestations. Participants of most studies were clinical diagnosed rather than pathology-proven according to kidney biopsy, which could cause misdiagnosis for non-diabetic renal diseases (NDRD) [9–13] and normoalbuminuric diabetic kidney disease [14]. Whether early-onset of type 2 diabetes mellitus (T2DM) is a risk factor for diabetic nephropathy (DN) progression remains unclear. Therefore, it makes sense to identify the role of early-onset T2DM in the progression of DN in a biopsy-confirmed cohort.

In this study, we investigated the clinical features, renal pathologic changes, and renal outcome in T2DM patients with early-onset and later-onset of T2DM in a biopsy-based cohort to elucidate the effect of early onset of T2DM on pathology and prognosis of DN, which could further indicate the role of early incidence of T2DM in well-confirmed DN patients.

## RESULTS

### Baseline clinical and pathologic characteristics

**Figure 1:**
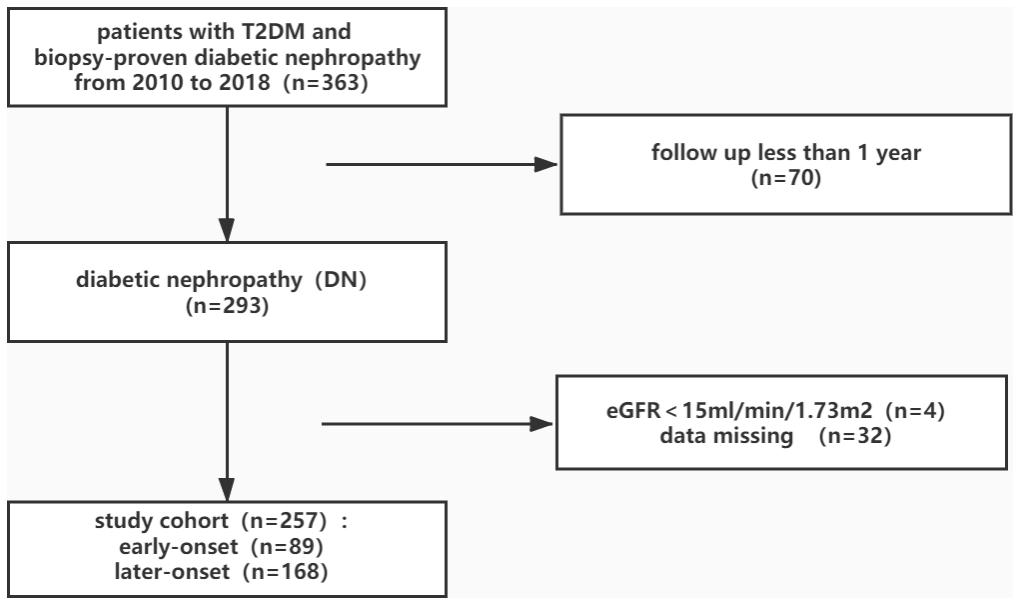
**Flowchart of study participants.**

A total of 257 subjects were enrolled in this study (Figure 1). At time of renal biopsy, the mean age of the patients was 51.48±9.28 years old and 69.6% (179 in 257) of them were men. 29.6% (76 in 257) of the patients had DM family history and the median T2DM duration was 8.00 (3.00,11.00) years. Hypertension presented in most of the patients (86.8%, 223 in 257), so were the use of insulin (73.2%, 188 in 257). Diabetic retinopathy (DR) was in 45.5% (117 in 257) of the total cohort. Chronic kidney disease (CKD) stage distribution were: stage1 28.8%, stage2 19.5%, stage3 39.3%, stage4 12.5%. The mean estimated glomerular filtration rate (eGFR) was 67.35±32.55 ml/min/1.73 m with the median 24 hours urine protein excretion of 4.30g (range 2.18-7.78 g). Baseline data were shown in Table 1.

**Table 1:** Baseline characteristics of enrolled patients.

	**Total** **n=257**	**Early-onset** **n=89**	**Later-onset** **n=168**	***p***
Men (%)	69.6	67.4	70.8	0.571
Age (years)	51.48±9.28	43.43±7.12	55.75±7.23	<0.001
Duration of diabetes (years)	8.00(3.00,11.00)	10.00(5.50,15.00)	5.00(2.00,10.00)	<0.001
Age of diabetes diagnosis (years)	43.59±10.32	32.82±5.58	49.29±7.25	<0.001
Family history (%)	29.6	43.8	22.0	<0.001
Hypertension (%)	86.8	85.4	87.5	0.635
Diabetic retinopathy (%)	45.5	58.4	38.7	0.003
Glycosylated hemoglobin (%)	7.58±1.99	7.52±2.13	7.62±1.92	0.754
Fasting blood glucose (mmol/L)	7.46(5.50,9.66)	7.52(5.19,9.70)	7.46(5.72,9.46)	0.775
Urinary protein excretion (g/day)	4.30(2.18,7.78)	4.44(2.53,9.08)	4.26(1.88,6.81)	0.184
eGFR (ml/min/1.73 m^2^)	67.35±32.55	70.34±36.13	65.77±30.48	0.286
Serum creatinine (μmol/L)	132.85±67.63	132.68±66.62	132.94±68.35	0.976
Serum uric acid (μmol/L)	389.04±82.91	387.94±84.17	389.62±82.49	0.877
Triglyceride (mmol/L)	1.80(1.26,2.42)	1.84(1.34,2.46)	1.79(1.22,2.42)	0.473
Cholesterol (mmol/L)	5.30±1.59	5.40±1.51	5.24±1.63	0.452
LDL-C	3.10±1.26	3.16±1.19	3.07±1.30	0.568
HDL-C	1.34±0.47	1.32±0.41	1.35±0.50	0.590
Insulin use (%)	73.2	80.9	69.0	0.041
RASI use (%)	79.8	79.8	79.8	0.967

eGFR, estimated glomerular filtration rate; RASI, Renin-Angiotensin-System inhibitor; LDL-C, low density lipoprotein cholesterol; HDL-C, high density lipoprotein cholesterol. Data are presented as the mean ± standard, median (quartile rang) or percentages.

*A two-tailed *p* < 0.05 was considered statistically significant.

In terms of the pathological glomerular classification, 5.1% were in class I, 23.7% in class IIa, 9.3% in class IIb, 45.9% in class III, 16.0% in class IV. The majority (97.3%, 250 in 257) of the patients had interstitial fibrosis and tubular atrophy (IFTA), arteriolar hyalinosis (88.7%, 228 in 257) and interstitial inflammation (92.6%, 238 in 257) pathological presentations (Table 2).

**Table 2:** Pathological features.

	**Total** **n=257**	**Early-onset** **n=89**	**Later-onset** **n=168**	*p*-value*
Glomerular classification				0.039
I	13	4	9	
IIa	61	19	42	
IIb	24	6	18	
III	118	38	80	
IV	41	22	19	
IFTA				0.088
0	7	3	4	
1	116	35	81	
2	103	34	69	
3	31	17	14	
Interstitial inflammation				0.606
0	19	8	11	
1	196	67	129	
2	42	14	28	
Arteriolar hyaline				0.019
0	29	8	21	
1	130	38	92	
2	98	43	55	

IFTA, interstitial fibrosis and tubular atrophy.

*Mann-Whitney test. A two-tailed *p* < 0.05 was considered statistically significant.

### Relationships between early-onset T2DM and clinicopathological features

Among the cohort, 34.6% (89 in 257) patients had their T2DM diagnosed less than 40 years old. Compared with later-onset group, patients with early-onset T2DM had longer diabetes duration and more insulin use. DR presented more frequently in early-onset group. DM family history presented more in early-onset other than later-onset patients.

Baseline eGFR, 24 hours urine protein excretion, serum uric acid, serum lipid and glucose level were comparable between early-onset and later-onset groups (Table 1).

As for pathological characteristics, the early-onset group presented more severe glomerular classification and arteriolar hyaline than those in later-onset group though the renal function was comparable between these two groups (Table 2). Early-onset group had thicker glomerular basement membrane (GBM) than later-onset group (890.73 ± 268.65nm vs 795.38 ± 274.80nm, *p* = 0.021). We also found that the age of T2DM diagnosis was negatively correlated with the arteriolar hyaline scores (r = -0.128, *p* = 0.04) and the thickness of GBM (r = -0.190, *p* = 0.008) in the total cohort.

### Early-onset of T2DM and renal outcome of DN

**Figure 2:**
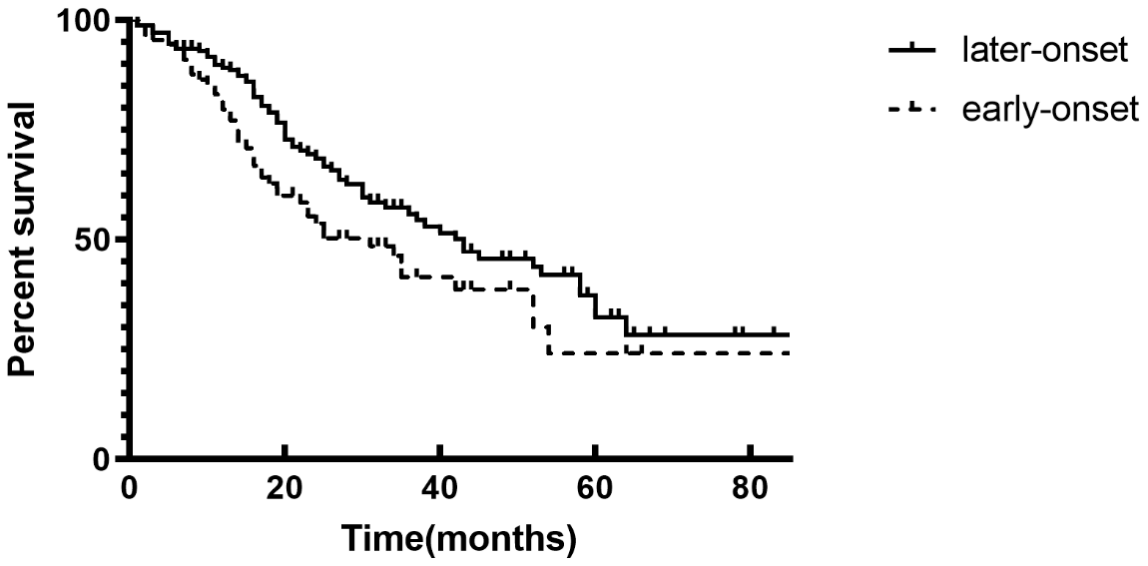
**Renal survival rate between patients in two groups with Kaplan–Meier survival analysis.** The event-free survival for end stage renal diseases (p = 0.031, log-rank test).

At the end of the follow-up, 47.1% of the patients reached the renal endpoint (121 in 257) with the median survival time of the total cohort was 38 months. The median survival was 31 months in early-onset group, while 43 months in later-onset. Log-rank test showed that patients with early-onset T2DM had worse renal prognosis than those with later-onset T2DM (*p* = 0.031) (Figure 2).

In univariate analysis of the Cox proportional model, early-onset of T2DM was significantly associated with worse renal outcome (hazard ratio [HR] 1.490, 95% confidence interval [CI] 1.032–2.149, *p* =0.033). In the case of confound factors, first we adjusted for gender, age, the duration of diabetes, serum glucose level, hypertension, serum lipid level, eGFR and urine protein excretion, early-onset group experienced higher risk of ESRD (HR 1.938; 95% CI 1.007–3.730, *p* = 0.047). We further adjusted insulin and rennin-angiotensin system inhibitor (RASI) use, early-onset of T2DM remained significantly associated with worse renal outcome for DN patients (HR 1.962; 95% CI 1.018–3.782, *p* = 0.044).

## DISCUSSION

In this retrospective biopsy-based study, we investigated the association of the age of T2DM onset and pathological characteristics as well as renal prognosis in biopsy-proven DN. We observed that compared with patients with later-onset T2DM, early-onset patients had more severe glomerular and vascular pathological lesions, though they were with comparable baseline renal function and proteinuria. Early-onset of T2DM was significantly associated with higher risk of ESRD in patients with DN. To our knowledge, this investigation may be the first biopsy-based study to clarify the relationship between the early-onset T2DM and DN pathological lesions, as well as the renal outcome. We confirmed the evidence to support that early-onset T2DM patients had worse renal prognosis in a biopsy-proven DN cohort.

Unlike type 1 diabetes mellitus often occurred in adolescents, we used to view T2DM as an old adult disease in the past. However, in recent years, T2DM is increasingly diagnosed in adolescents and young adults [15, 16]. In some countries, the prevalence of T2DM has even exceeded T1DM among young people [5, 17]. Compared with those with later-onset T2DM, early-onset patients would have longer lifetime exposure to hyperglycemia, which could cause long-term diabetic complications. Some previous studies [18–20] have confirmed that patients with early-onset T2DM had higher prevalence of microvascular complications than those with later-onset, including DN. Our data showed that in patients with biopsy-proven DN, those with early-onset T2DM had higher risk of ESRD than later-onset ones though their renal function and proteinuria were similar at the time of renal biopsy, which was coincident with several earlier studies. Liu et al [8] found that in a 1189 Asian T2DM cohort, patients with early-onset DM had higher risk of progressive CKD in both albuminuria and renal function deterioration. Another study [21] enrolled 1111 individuals with T2DM and clinical diagnosed DN indicated that patients with early-onset had higher prevalence of ESRD and early-onset was an independent risk for ESRD after adjusted for traditional risk factors. However, patients with DN enrolled in these studies were all clinical diagnosed, which could cause misdiagnosis for NDRD. Our study added evidence to support the role of early-onset T2DM in DN among biopsy-confirmed cohort, which might clarify the direct renal complication caused by DM.

We also investigated the association between the early-onset of T2DM and renal pathological lesions in patients with DN. With the comparable eGFR and proteinuria (Table 1), early-onset group presented more severe glomerular classification and arteriolar hyaline as well as thicker GBM than those in later-onset group, which indicated worse renal pathological presentation (Table 2). Although hypertension, the serum glucose and lipid levels were similar between groups, patients with early-onset had longer diabetes duration and more insulin use, which indicated deteriorated metabolic dysfunction and caused worse pathological presentation. Our previous study [11, 22] showed that patients with severe glomerular classification and thicker GBM had higher risk of ESRD, which could partly explain why early-onset group had worse renal outcome.

The mechanisms of the association between early-onset of T2DM and DN progression remined unclear. Early-onset group had longer diabetes duration than later-onset group in our cohort, which was suggested to be related to more severe diabetic complications in some previous studies [23, 24]. However, we found that after adjusted for diabetes duration and other typical risk factors, early-onset of T2DM remained a significant predictor for ESRD in patients with DN, which suggested a sole role of early-onset in DN progression rather than resulted from longer diabetes duration. Druet et al [25] found that second phase of nutrient-induced insulin secretion might reduce faster in early-onset T2DM than elder, which might be related to abnormal tubular-glomerular feedback and cause DN progression. As TODAY Study indicated, patients with early-onset T2DM had accelerated decline of β-cell function than those with later-onset [26]. We also found more insulin use in early-onset group in this cohort. Deterioration in β-cell function meant untimely and more insulin use, which could cause chronic complications. In our study, early-onset group presented higher proportion of DM family history, which was similar with previous studies [27, 28]. A past study of our group also showed that patients with DN and family history of DM had more aggressive renal disease and rapidly falling in eGFR [29]. Other unknown factors remain further exploration.

This study has several limitations. First, we could not exclude all possible confounding factors for the complexity of DN. Then, the natural history of T2DM is quite complex, we could only trace the age they were diagnosed as T2DM rather than the precise onset time of T2DM. So the clinical silence of T2DM might affect our conclusion. Finally, it was not routine for patients with DN to undergo renal biopsy, so the sample size was limited and the patients had relatively advanced DN, which inevitably contained inherent selection bias. Therefore, it is necessary to confirm this hypothesis in large-scale biopsy cohort.

## CONCLUSIONS

In conclusion, although with the comparable renal function and proteinuria, patients with early-onset T2DM and DN had worse renal pathological changes than those with later-onset. Early-onset of T2DM might be an important predictor of ESRD for patients with DN, which called more attention to early supervision and prevention for patients with early-onset T2DM and DN for the higher risk of DN progression.

## MATERIALS AND METHODS

### Patients

Patients with T2DM who underwent renal biopsy from 2010-2018 in West China Hospital, Sichuan University were enrolled in this study. Included patients were with biopsy-confirmed DN and followed up for at least one year. Exclusion criteria were as follows: comorbid with systemic or nondiabetic renal diseases; presence of ESRD at the time of renal biopsy (Figure 1). Diagnosis of DM was based on the standard of the American Diabetes Association [30]. General indications for kidney biopsy were as follows: absence of DR, sudden onset of overt proteinuria or rapidly falling of eGFR, presence of active urinary sediment. The pathology diagnosis of DN was evaluated in 2010 Renal Pathology Society classification [31] and confirmed by at least two pathologists.

A total of 257 subjects were enrolled in this study (Figure 1). These patients were divided into two groups according to their age of T2DM diagnosis. We defined early-onset of T2DM group as less than 40 years and 40 years or older as later-onset group, just like some previous studies [21, 32]. The endpoint of patients was the progression to ESRD (eGFR < 15 ml/min/1.73 m^2^, or the initiation of renal replacement therapy). Everyone who enrolled in the study agreed and signed informed consent. The study was approved by the ethics committee of West China Hospital, Sichuan University.

### Clinical and renal pathological features

Clinical data in baseline was collected by interview and anthropometry at the time of kidney biopsy, including age, sex, duration of diabetes, family history, blood pressure, and medication history. Blood and urine samples were collected and biochemical parameters were measured by a biochemistry auto-analyzer (Cobas Intera 400 Plus, Roche, Basel, Switzerland), including serum uric acid, creatinine, triglycerides, cholesterol, fasting glucose, glycated hemoglobin (HbA1c), hemoglobin (Hb), and 24-hour urine protein excretion. The eGFR was calculated using the Modification of Diet in Renal Disease equation. DR was diagnosed by optical coherence tomography (OCT) and fundus color photography according to 2002 Global Diabetic Retinopathy Project Group standard [33]. Renal biopsy was obtained by needle and processed for light microscopy, electron microscopy and immunofluorescence to evaluate kidney pathological characteristics. Each sample was examined by at least two pathologists from the same group in West China Hospital. Pathological features were categorized for the classification of the Renal Pathology Society [31].

### Statistical analysis

Statistical analyses were performed with SPSS 22.0 (IBM, USA). Variables are presented as the mean ± standard deviation, median with interquartile range or proportion where appropriate. Differences between groups were assessed with the t-test for normally distributed variables, non-parametric test for skewed distributed variables and with the Chi-square test for categorical variables. Correlation testing was used to analyze the associations between early-onset of T2DM and DN. Kaplan-Meier analysis was for renal survival by log-rank test. Multivariate-Cox analysis was applied for exploring the relationship between early-onset of T2DM and renal outcome. *p* value < 0.05 was considered statistically significant.
